# Greater Skeletal Gains in Ovary Intact Rats at Maturity Are Achieved by Supplementing a Standardized Extract of *Butea monosperma* Stem Bark that Confers Better Bone Conserving Effect following Ovariectomy and Concurrent Treatment Withdrawal

**DOI:** 10.1155/2013/519387

**Published:** 2013-04-27

**Authors:** Kamini Srivastava, Kainat Khan, Abdul M. Tyagi, Mohd. P. Khan, Dinesh K. Yadav, Ritu Trivedi, Rakesh Maurya, Divya Singh, Naibedya Chattopadhyay

**Affiliations:** ^1^Endocrinology Division and The Center for Research on Anabolic Skeletal Targets in Health and Illness (ASTHI), CSIR, Central Drug Research Institute, Sector 10, Jankipuram Extension, Sitapur Road, Lucknow 226021, India; ^2^Division of Medicinal and Process Chemistry, CSIR, Central Drug Research Institute, Sector 10, Jankipuram Extension, Sitapur Road, Lucknow 226021, India

## Abstract

With a longitudinally designed study, we tested whether an acetone soluble fraction (ASF) from the stem bark of *Butea monosperma* resulted in maximizing bone gain in rats during growth and maturation and thus protected against osteopenia following ovariectomy (OVx) with concomitant treatment withdrawal. Female rats at weaning were given ASF (100 mg/kg/d) or vehicle for 12 weeks, and baseline skeletal parameters (micro-CT) and total plasma antioxidant status (TAS) were measured. At this stage, one group was OVx and the other group was sham operated. Vehicle group (untreated) after OVx was given E2 or continued with vehicle (OVx control). ASF group after OVx was given vehicle (ASF withdrawn, ASFW). After another 12 weeks, all groups were killed and various skeletal parameters were determined. ASF resulted in substantially better skeletal parameters and higher plasma TAS over control at maturity. Rats treated with ASF before OVx had reduced rates of bone loss compared to OVx control. Twelve weeks after OVx, the ASFW group exhibited better trabecular microarchitectural preservation, bone turnover profiles, increased cortical deposition, and biomechanical strength over the OVx control, and the effects were comparable to OVx + E2 group. ASF supplementation during skeletal growth could maximize bone accrual and could confer increased resistance to post-OVx osteopenia despite treatment withdrawal.

## 1. Background

Bone mass after menopause is critically dependent on the bone quantity attained during young adulthood and the rate at which bone is lost afterwards. The highest bone amount, peak bone mass, has a vital role in bone health. It is suggested that one standard deviation increase in peak bone mass could cut down the risk of fracture by as much as 50%, since individuals who accrue a high peak bone mass may preserve a higher BMD throughout their lives [1]. Thus, preventive approaches against osteoporosis may aim at either increasing the peak bone mass or diminishing the rate of bone loss during aging. 

Peak bone mass is a combination of multiple factors, with genetic influence being central [2, 3], although several environmental and nutritional factors of varying importance regulate bone gains during childhood and adolescence [4]. Women from East Asian countries are about half as likely as Caucasian women to experience a hip fracture [5, 6]. One possible explanation is that lifelong habitual consumption of soy isoflavones exerts a protective effect in postmenopausal women, either by increasing peak bone mass [7] or by decreasing the rate of bone loss after menopause [8]. So far, studies reports inconsistent effect of soy isoflavones on bone. For example, a randomized trial data showed that isoflavone exposure had no effect on bone density among relatively young (21–25 years old) women, most of whom were white [9]. This finding is consistent with a study on premenopausal cohort indicating that differences in isoflavone consumption are not associated with variation in bone mass [10]. Similarly, studies using rats or mice with intact ovarian function failed to find significant increases in bone mass after soy or isoflavone supplementation [11]. Studies on premenopausal cynomolgus monkeys showed that chronic consumption of high-isoflavone soy protein did not significantly alter spinal BMD and resorption marker compared with the control [7]. Together, these reports question the ability of soy isoflavones in augmenting bone mass in premenopausal or under active ovarian condition and leave the scope for finding a better alternative.

*O*-methoxy substitutions of free phenolic hydroxyl groups of the most abundant soy isoflavones (genistein and daidzein) enhance the lipophilicity, metabolic stability, and uterine safety, thus improving pharmacokinetic/metabolic stability profiles of genistein and daidzein and, consequently, enhance the pharmacodynamic effect (*in vivo* potency) [12, 13]. In our phytopharmacological evaluation program, aimed at discovering effective alternative strategy for reducing the risk of developing postmenopausal osteopenia, we showed that a standardized fraction (an acetone soluble fraction, ASF) made from the stem bark of *Butea monosperma* contained four methoxyisoflavones: cajanin (7-methoxy genistein), medicarpin (a methoxypterocarpan with cyclized genistein ring structure), isoformononetin (7-methoxy daidzein), and cladrin (3′4,-dimethoxy daidzein) at percent concentration of 0.061, 0.019, 0.007, and 0.003, respectively [14]. Each one of these, when administered to female rats for four weeks after weaning resulted in increased BMD, bone strength, and bone formation rate with varying efficacy. *In vitro*, all four compounds stimulated osteoblast function more potently than genistein and daidzein by different modes of action [15–17]. These observations prompted us to hypothesize that the presence of these methoxyisoflavones in the ASF could synergistically augment peak bone mass accrual in female rats at maturity that will confer a superior bone conserving ability after surgical menopause (due to ovariectomy, OVx) even as the treatment is withdrawn.

Accordingly, the present study used a longitudinal design in which female rats at weaning were treated with either vehicle or ASF by gavage for 12 weeks, followed by concurrent termination of treatment and induction of estrogen deficiency by OVx. Since ASF has been reported to induce new bone formation and prevent OVx-induced bone loss at 100 mg/kg dose [14], this dose was used in the present study. Skeletal responses by the treatment and its abandonment were evaluated by BMD, trabecular microarchitecture, biomechanical strength, and bone metabolic markers. Being a phytoestrogen, the bone conserving effects of ASF upon treatment withdrawal in OVx rats were compared with OVx animals administered with 17-beta estradiol (E2) at a pharmacological dose that was comparable to that recommended for estrogen replacement therapy given to postmenopausal women for the protection of bone loss. 

## 2. Material and Methods

### 2.1. Reagents and Chemicals

E2, calcein, and tetracycline were purchased from Sigma-Aldrich (St. Louis, MO, USA). Osteocalcin (midportion) and fragments of type 1 collagen (CTx) ELISA kits were purchased from Immunodiagnostic Systems Ltd. (Tyne and Wear, UK). Total antioxidant status (TAS) Kit was purchased from Randox Laboratories Ltd. (Crumlin Co., Antrim, UK). The stem bark of *Butea monosperma* (Lam.) Taub. was collected from Lucknow, Uttar Pradesh, India, in the month of September 2008. The collection and authentication were made by Botany Division of Central Drug Research Institute. Voucher specimen (no. 1020) is kept in the herbarium of the institute. The acetone soluble fraction (ASF) was synthesized from total ethanolic extract of stem bark of *B. monosperma* [14].

### 2.2. Experimental Design

The study was conducted in compliance with the standards mentioned by Institutional Animal Ethical Committee (IAEC) at Central Drug Research Institute; the CPCSEA (Committee for the Purpose of Control and Supervision on Experiments on Animals) registration number of the IAEC is 34/1999. Female Sprague-Dawley rats were obtained from the National Laboratory Animal Centre, CSIR-CDRI. All rats were housed in a room maintained at 25°C in 12 : 12 hour light/dark cycles. Standard laboratory rodent chow diet devoid of soy protein and water was provided ad libitum.

**Figure 1:**
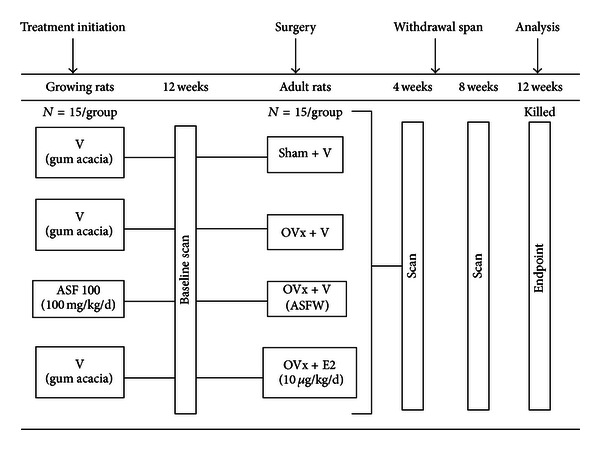
Experimental design. Three-week-old (weaned) female SD rats were given either vehicle (daily/p.o.) or ASF (100 mg/kg/d/p.o.) for 12 weeks (baseline). At baseline, BMD and microarchitectural parameters were recorded and serum TAS was measured. Rats were then either sham operated or OVx and were given either vehicle or E2 (10 *μ*g/kg/d/p.o.) for another 12 weeks (endpoint). BMD and microarchitectural parameters were recorded at 4- and 8-week following OVx. After 12-week following OVx (endpoint), all animals were killed; blood, urine (24 h), tibia, femur, and vertebrae were harvested and analyzed.

Figure 1 summarizes the study plan and experimental groups. Sixty recently weaned female Sprague-Dawley rats (3 weeks old; 20 ± 5 g each) were randomly assigned to two groups: 1% gum acacia (vehicle) group (*n* = 45), and 100.0 mg/kg/d ASF administered group (*n* = 15) [14]. Treatment was given by daily oral administration for a period of 12 weeks. After 12 weeks, which served as baseline, cortical and trabecular bone parameters were determined by live imaging of rat using micro-CT scans. For the measurement of total antioxidant status in serum, blood was drawn from retro-orbital plexus.

Subsequently, all the rats were ovariectomized (OVx). ASF treated rats after OVx and concurrent treatment withdrawal (vehicle replaced ASF treatment) referred as ASF withdrawal (ASFW; *n* = 15). Untreated (vehicle) group after 12 weeks was either sham operated or ovariectomized and was further divided into three groups as follows: ovary intact sham group given vehicle (sham + veh; *n* = 15), OVx rats given vehicle (OVx + veh; *n* = 15), and OVx rats given 10.0 *μ*g/kg/d E2 by oral gavage (OVx + E2; *n* = 15). E2 dose was based on previous reports [18]. The animals were housed for another 12 weeks after which they were killed. Live animal imaging was done at 4 weeks and 8 weeks after OVx.

After an additional 12 weeks (endpoint) all groups were killed (Figure 1). On the day of killing, femur, tibia, and lumbar vertebrae were collected; bones were cleaned of muscles and stored in 70% isopropanol at 4°C until further analysis. Urine and serum samples were harvested following 24 hr starvation. Body weight of each animal was taken before the start of the experiment, at baseline and at the endpoint.

For determination of new bone formation *in vivo*, a previously published protocol was followed [19]. Briefly, each rat received intraperitoneal injection of fluorochromes tetracycline (20 mg/kg) on 30 days and calcein (20 mg/kg) on 28 days (12 week) before autopsy.

### 2.3. Micro-CT Scans

*In vivo* micro-CT scans of whole animal were obtained after 12-week treatment (baseline) and at 4 and 8 week following OVx and withdrawal after anesthetizing rats with ketamine (90 mg/kg) and xylazine (10 mg/kg) during the scan, which lasted about 20 min. *Ex vivo* scans of dissected tibia, femur, and vertebrae at the endpoint were also performed using the Sky Scan 1076 CT scanner (Aartselaar, Belgium) as described before [20]. Reconstruction of scanned bones was carried out using the SkyScan NRecon software. 

Bone length was determined from the rendered 3D images in CTAn software by drawing scale bar to individual bone image, for femur total length was defined as distance from greater trochanter to the edge of the femoral condyles while total length of tibia was from medial condyle to medial malleolus. The growth plate was isolated from the surrounding bone tissue in the micro-CT images by manual segmentation of 2D slices of sagittal images. The segmented sections were then reconstructed to render 3D images. From these images, growth plate height was measured using data viewer software. To analyze trabecular region, ROI was drawn at a total of 100 slices in the region of secondary spongiosa situated 1.5 mm away from the distal border of growth plate excluding all primary spongiosa and cortical bone. For cortical bone analysis, 350 serial image slides were discarded from growth plate to exclude the trabecular region, and 200 consecutive image slides were selected and quantification was done using CTAn software. Various trabecular parameters (3D) and cortical parameters (2D) were analyzed by following previously published protocols [21]. Using micro-CT scans, cortical and trabecular BMD of femur and tibia were determined from the VOI made for cortical and trabecular region, respectively. For calibration, the hydroxyapatite phantom rods of 2 mm of diameter with known BMD (0.25 g/cm^3^ and 0.75 g/cm^3^) were employed. For each analysis, the estimated mineral density of the bone tissue was determined based on the linear correlation between micro-CT attenuation coefficient and bone mineral density [22].

### 2.4. Bone Mechanical Strength

Bone mechanical strength was examined by 3-point bending strength of femur middiaphysis and compressive strength of the fifth lumbar vertebrae using Bone Strength Tester Model TK-252C as we reported earlier [20, 21]. The load-displacement curves generated were used to calculate the ultimate load (N), stiffness (N/mm), and energy to failure (mJ). 

### 2.5. Fluorochrome Labeling and Bone Histomorphometry

Methods followed our previously published protocol with some modifications [16, 19]. Briefly, cross sections (50 *μ*m) of distal regions of undecalcified femur diaphysis of each rat embedded in acrylic material were obtained using an IsoMet Low Speed Bone Cutter (Buehler, Lake Bluff, IL, USA). Histomorphometric measurements of MS/BS (%); MAR (*μ*m/day); BFR/BS (*μ*m^3^/*μ*m^2^/day) of cortical bone were performed according to standardized protocols of the American Society for Bone and Mineral Research [23]. To correct the label escape error, the level of bone surface actively mineralizing was calculated as the sum of the double label surface plus half of the single label surface [24].

### 2.6. Measurement of Total Antioxidant Status and Bone Relevant Serum and Urinary Markers

Measurement of total antioxidant status (TAS) in serum sample (baseline) was carried out using ABTS+ (2,2′-azidodiethylbenzothiazolin sulphonate) radical formation kinetics (Randox Laboratories Ltd.). The presence of antioxidants in serum suppressed the bluish-green staining of the ABTS + cation, which was proportional to the antioxidant concentration. Kinetics was measured at 600 nm.

On the basis of our previously published protocols [25], serum osteocalcin (midportion), urinary CTx levels were determined by enzyme-linked immunosorbent assay kits purchased from Immunodiagnostic Systems Inc. by following the manufacturer's protocols. 

### 2.7. Statistics

Data are expressed as mean ± SEM. The data obtained in experiments with multiple treatments were subjected to one-way ANOVA followed by post hoc Newman-Keuls multiple comparison test of significance using GraphPad Prism 5 software. An unpaired form of Student's *t-*test was used to compare base line data (after 12-week treatment) and endpoint data (12 weeks following OVx). 

## 3. Results

### 3.1. Effect on Body Weight, Serum TAS, and Appendicular Skeleton of Rats at Baseline

ASF (100 mg/kg/day dose) treatment to weaned female rats for 12 weeks was well tolerated and resulted in no significant change in body weight when compared to the vehicle group (control). Plasma TAS was 57% higher in the ASF group compared to the control. ASF group had 5%  *P* < 0.01 and 8%  *P* < 0.05 increases, respectively, of femur and tibia length compared with the control. In comparison to controls, ASF group also had 9% increase in the height of growth plates at femur and tibia (Table 1). 

The effect of ASF treatment on bone mass and microarchitectural parameters in cancellous and cortical bone are described in Table 1. Micro-CT analysis showed that supplementation of ASF for 12 weeks significantly increased the BMD of both femur and tibia compared with the control. Increase was more robust at the cancellous sites (25% in femur epiphysis and 32% in proximal tibia over the vehicle group). BMD at femur cortical sites was 5% higher in ASF over placebo group. Assessment of cortical bone at the femur mid-diaphysis showed a significant increase in bone area (B.Ar), cortical thickness (Ct.Th), and mean polar moment of inertia (MMI) in ASF treated group over the control, while periosteal area (T.Ar) and marrow area (Ma.Ar) were not different between the two groups. 

At femur metaphysis, ASF treatment resulted in significant increase in bone volume fraction (BV/TV), trabecular number (Tb.N), and connectivity density (Conn.D) compared to control, while trabecular separation (Tb.sp), trabecular pattern factor (Tb.pf), and structure model index (SMI) were reduced. Trabecular thickness (Tb.Th) and degree of anisotropy (DA) were not different between the groups. Tibial data at metaphysis also showed substantial gain in BV/TV, Tb.N, and Conn.D and fall in Tb.sp, Tb.pf, and SMI in ASF treated group when compared to control. 

### 3.2. Effect of OVx and Concurrent Treatment Withdrawal after 8 weeks

**Figure 2:**
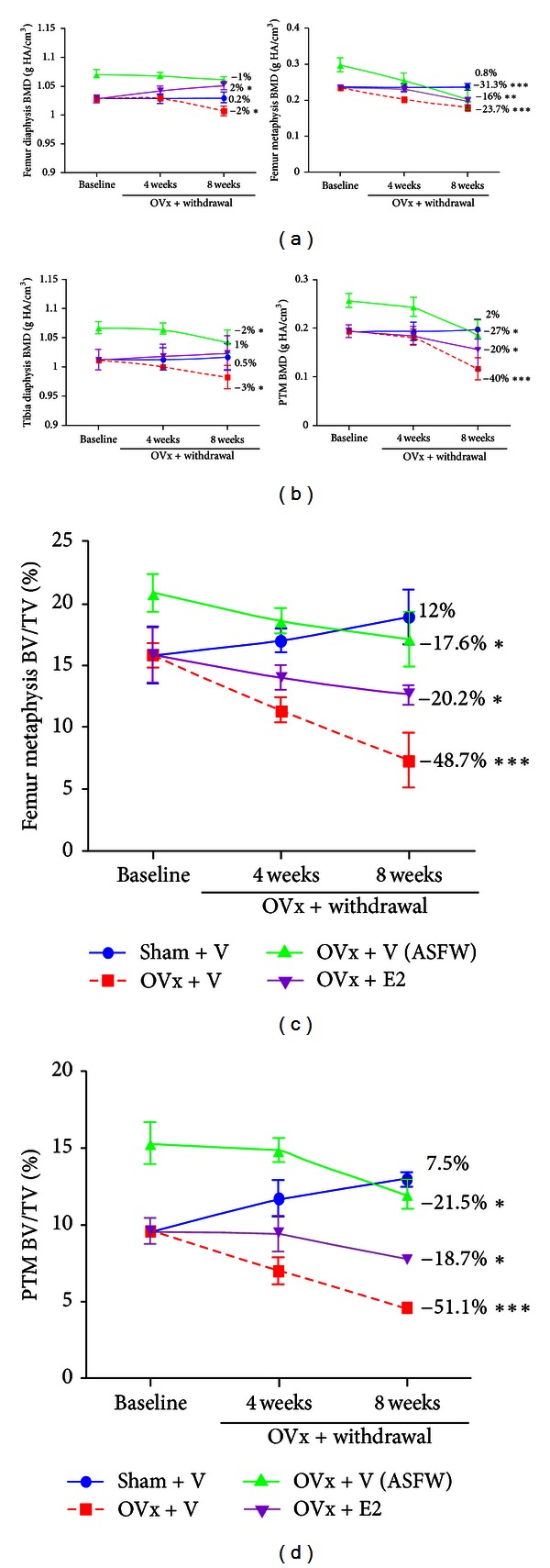
Effect of OVx and concurrent ASF withdrawal (ASFW) on appendicular bones. (a) BMD at femur diaphysis and metaphysis, (b) BMD at tibia diaphysis and metaphysis, (c) BV/TV at femur metaphysis, and (d) BV/TV at proximal tibia metaphysis. Mean percent changes from baseline were calculated and shown (*n* = 15 rats/group). *P < 0.05, **P < 0.01, and ***P < 0.001 compared to baseline.

The results of *in vivo* BMD measurements at cortical and trabecular sites of femur and tibia among the various groups are presented in Figures 2(a) and 2(b). After 8 weeks following OVx, OVx + veh group showed reduction in BMD by 2% in femur diaphysis and 3% in tibia diaphysis compared to their respective baseline values (both *P* < 0.05 versus baseline). BMD in OVx + E2 group increased marginally, 2% in femur (*P* < 0.01 versus baseline), while it remained the same in tibia. ASFW group preserved BMD in the femur diaphysis; while reduced by 2% in tibia diaphysis (*P* < 0.05 versus baseline). The trabecular BMD in OVx + veh group after 8 weeks was reduced significantly, 23.7% in femur metaphysis and 40% in tibia metaphysis (both *P* < 0.001 versus baseline). Ovx + E2 group had significant lesser BMD loss, 16% in femur (*P* < 0.01 versus baseline) and 20% in tibia (*P* < 0.05 versus baseline). ASFW group showed 31% (*P* < 0.001 versus baseline) and 27% (*P* < 0.05 versus baseline) BMD reduction, respectively, at femur metaphysis and tibia diaphysis.

Figure 2(c) showed that in comparison to the baseline, the ovary intact (sham) group after 8 weeks showed a tendency to increase in BV/TV at femur and tibia cancellous bones although statistically nonsignificant. OVx controls exhibited a sharp decline in BV/TV at 8 weeks, 48.7% in femur and 51.1% in tibia (both *P* < 0.001 versus baseline). OVx + E2 group had a much attenuated fall, 20.2% and 18.7% respectively in femur and tibia (*P* < 0.05 versus baseline). ASFW group also exhibited a sluggish declining pattern, 17.6% and 21.5%, respectively, in femur and tibia (*P* < 0.05 versus baseline). At 8 weeks, BV/TV in ASFW corresponded to the baseline value of the sham group.

Table 2 shows percent change from baseline in all the trabecular bone parameters of the appendicular skeleton. In the OVx + veh group, all parameters except Tb.Th and DA showed deterioration in both femur and tibia. ASFW and OVx + E2 also showed significant deterioration in all parameters at both sites except Tb.Th; however, the extent of deterioration was significantly less than the OVx + veh group. When comparisons were made between the ASFW and OVx + E2 groups, the former had a better trabecular profile with increased femoral Tb.N, tibial Tb.Th, decreased femoral and tibial Tb.sp, and Tb.pf, and no change in other parameters.

### 3.3. Effect of OVx and Concurrent Treatment Withdrawal on Bone Parameters after 12 weeks (Endpoint)

At the endpoint, body weight of OVx rats treated with vehicle for 12 weeks weighed 30% more than the sham (*P* < 0.001). E2 treated OVx rats weighed significantly lesser than the OVx + veh group. There was no difference in the weight of OVx + veh and ASFW groups.

Serum OCN and urinary CTx were increased by 132% and 145%, respectively, in the OVx + veh group compared with sham (both *P* < 0.001). Both parameters were significantly reduced in OVx + E2 and ASFW groups compared with OVx + veh. However, OCN was higher in the ASFW group compared with OVx + E2 group.

Micro-CT was performed on excised bones to compare various parameters between the groups at the endpoint. When BMD measurement of femur metaphysis, tibia proximal metaphysis, and L5 vertebra was compared with the sham, it showed remarkable fall in all OVx groups. OVx + E2 group had significantly higher BMD at all three sites compared to OVx + veh group. On the other hand, ASFW group had maintained the gain attained during treatment to certain extent, such that it has significantly higher BMD when compared to OVx + veh, while BMD was not different between OVx + E2 and ASFW group.

Femoral trabecular data showed that compared with the sham, all OVx groups resulted in the deterioration of the trabecular parameters represented by reduced BV/TV, Tb.N and Conn.D and increased Tb.sp, Tb.pf, and SMI (Table 3). Repletion of E2 to OVx rats had marginally increased BV/TV and Tb.N and decreased Tb.sp, Tb.pf, and SMI compared to OVx + veh group, while Conn.D was unchanged. Compared to OVx + veh group, ASFW group showed increased BV/TV, Tb.N, and Conn.D and decreased Tb.pf and SMI, while Tb.sp was unchanged. 

Similar deterioration in trabecular profile in the OVx groups at the tibia metaphysis was observed. ASFW group or OVx + E2 group resulted in significantly increased BV/TV due to increase in Tb.N and also increased Conn.D, but decreased Tb.sp, Tb.pf, and SMI when compared to OVx + veh group. None of these parameters were different between OVx + E2 and ASFW groups.

L5 vertebra exhibited enormous trabecular deterioration in all three groups 12 weeks following OVx (Table 3). OVx + veh group showed a significant fall in BV/TV, Tb.Th, Tb.N, and Conn.D and increase in Tb.sp, Tb.pf, SMI, and DA as compared to the sham. In comparison to OVx + veh group, OVx + E2 or ASFW showed increased BV/TV, Tb.Th, and Conn.D and decreased Tb.sp, SMI, and DA. Except for SMI, which was reduced more in the ASFW group than E2 supplemented group, no significant difference in other parameters was observed between the ASFW and OVx + E2 groups.

We next studied the effect of various treatments on vertebral strength by compression test. Compared to the sham group, L5 of OVx + veh group showed significant reduction in ultimate load, energy to failure, and maximum stiffness. All parameters between the sham and ASFW groups were comparable whereas those between the OVx + E2 and sham, energy to failure and maximum stiffness were not different but ultimate load was higher in the sham group. 

Dynamic histology of femur diaphysis was compared between the groups (Table 4). Mineralizing surface per bone surface (MS/BS), mineral apposition rate (MAR), and bone formation rate per bone surface (BFR/BS) were significantly reduced in the OVx + veh group compared with the sham. MS/BS was not different between the sham and ASFW groups; however, MAR and BFR/BS in the ASFW were lower than the sham but higher than the OVx + veh group. All parameters were comparable between the OVx + veh and OVx + E2 groups.

2D-micro-CT measurements at the site of femur mid-diaphysis (Table 4) at the endpoint showed that relative to sham group, OVx + veh had decreased B.Ar and Ct.Th while Ma.Ar and MMI were increased, and T.Ar was unaltered. Compared to OVx + veh group, OVx + E2 group had increased B.Ar and decreased endosteal area while Ct.Th, T.Ar and MMI were comparable. In comparison to OVx + veh group, ASFW group showed a significantly higher B.Ar and Ct.Th but endosteal area and MMI were reduced. Between ASFW and OVx + E2 groups, T.Ar, and marrow area were not different; however, Ct.Th was increased and MMI was decreased in ASFW group.

A three-point bending test was used to assess bone strength at the femoral midshaft (Table 4). There was evidence for decreased bone strength in the OVx + veh group compared with the sham operated group, with a significant decrease found in ultimate load, energy to failure, and stiffness. The increased femoral bone mass in ASF treated rats prior to OVx and withdrawal was translated into significant increases in all three parameters at the endpoint when compared with OVx + veh. Moreover, these parameters were comparable in ASFW and OVx + E2 groups. 

## 4. Discussion

There is a continuing debate, whether the attainment of peak bone mass or bone loss is the most crucial factor when it comes to the mechanism of developing osteoporosis [26–28]. The most important findings from this study are (1) treating weaned female rats with ASF, rich in methoxyisoflavones until the attainment of a mature skeleton resulted in better cortical and trabecular bone mass and architecture and (2) subsequent OVx with concurrent withdrawal of ASF treatment attenuated the rate of trabecular loss when compared to E2 supplemented group, which exhibited significant bone conserving effect on a par with E2. 

Increased consumption of fruits and vegetables, soy-based diets, dietary supplementation with antioxidant vitamins (C and E), and phytopreparations rich in polyphenolic antioxidants are considered beneficial for health in general. With respect to skeleton, antioxidants not only counteract the increased production of osteoclasts due to reactive oxygen species (ROS) action on the precursor cells [29] but also enhance osteoblast longevity, which is reduced by ROS action [30, 31]. Together, these events establish the causal link between the oxidative stress on the body and bone loss. Our data showed that in comparison to the control, ASF treatment resulted in a 57% increase in plasma TAS. Presence of polyphenolic compounds, including cajanin, medicarpin, cladrin, and isoformononetin in ASF appeared to increase BMD and impart superior microarchitectural indices of bone at the baseline as each of these compounds improved bone mass in growing female rats, although possible contribution by other phytochemicals in the extract in increasing TAS cannot be ignored. Whether the superior skeletal outcome by ASF at the baseline is primarily due to the antioxidant effect of the extract or reduced estrogen elimination due to competition with the hepatic glucuronidation pathway of E2 by the flavonoids [32] present in ASF as reported in case of some flavonoids or both remain to be determined. Because reduction in E2 metabolism would translate to an amplification of estrogenic response [33], ASF is unlikely to cause such an effect as it has no uterine estrogenicity in young and adult rats [14]. Nonetheless, a greater modeling directed bone growth via the ER-dependent or independent pathways in osteoblasts by the constituents of ASF coupled with its antioxidant effect appears to be the mechanism leading to positive skeletal effects of ASF over the placebo group at baseline (ovary intact).

In rats, a skeletal growth spurt for the first 5 weeks is followed by a sluggish phase giving way to skeletal maturity, which is attained by 11.5–13 weeks [34]. ASF treatment of rats from 3 to 16 weeks covered both skeletal growth and maturity phases and resulted in increased lengths and growth plate heights of long bones compared to control. Linear bone growth results from chondrocytes at the growth plate that undergo cell division in the proliferating zone and cell differentiation in the hypertrophic zone [34]. Presently, we have no data to conclude whether increased growth plate height by ASF was due to its effect on proliferating or hypertrophic zone. Besides length gain, cortical bones during the growth also increase in width by apposition of subperiosteal bone [27]. ASF treatment resulted in significant increases in the cortical BMD as well as width of femur and tibia, as shown by larger cross-sectional area and a thicker cortex compared with control rats. More cortical parameters in the femur were positively modulated by ASF than the tibia, suggesting its preference for skeletal site. Net bone gain at both sites were not located in the endosteal surface as T.Ar and B.Ar were comparable between the ASF and placebo groups, which further suggested that the effect of ASF was unlike E2 as the hormone is known to favor endosteal deposition [35]. Taken together, these data suggested that ASF promoted accrual of cortical bone.

The effect of ASF treatment on connectivity measures and trabecular geometry was better than the placebo group. This gain in cortical bone and improvement in microarchitectural measures are related to the increased rate of bone elongation. The extent of changes in various parameters between femur and tibia by ASF treatment was largely comparable. Increase in Tb.N might have arisen from stimulation of new trabeculae from the growth plate. Conn.D reflects trabecular bone connectivity, which is a structural property of cancellous bone that affects cortical bone strength and make the bone less fracture prone [36]. Generally, bone with a well-connected trabecular bone network protects cortical bone by diminishing the retention and/or buildup of local damage by diffusing it instantly through the trabecular bone network [37]. Indeed, studies have demonstrated that bones with a well-connected trabecular bone have increased strength [38]. Structural and geometric indices, including SMI and Tb.pf, suggest that ASF treatment would render the cancellous bones better suited over the placebo group to resist fracture.

Trabecular bone is readily lost due to E2 deficiency that characterizes postmenopausal bone loss [39], which is apparent from the greater decline in trabecular over cortical BMD at femur and tibia of the OVx + veh group. Fall in the trabecular bone volume fraction in OVx control was nearly 50% in both femur and tibia cancellous bones from their baseline levels after 8 weeks following OVx that accompanied dramatic deterioration in trabecular profiles (considering all trabecular parameters). Compared to baseline, reduction in the trabecular bone volume fraction in ASFW was remarkably suppressed, ~20% at both sites, which was comparable to E2 supplemented group, and at both appendicular sites, ASFW had a better cancellous architecture profile over the E2. These results suggest a robust dampening of trabecular bone loss by ASF pretreatment after 8 weeks following OVx. Moreover, advantage of bone gain by ASF treatment at the baseline was quite substantial given that ASFW group at 8 weeks following OVx had BMD and trabecular bone volume fraction largely comparable to the sham + veh.

Twelve weeks following OVx (endpoint), femur and tibia of OVx control rats showed a significant reduction in trabecular BMD, bone volume, and Tb.N, as well as an increase in Tb.sp, a series of changes that resulted in a decreased connectivity among trabeculae and led to increased Tb.pf value. Apart from poor connectivity, geometric measure showed worsening impact in OVx due to higher SMI (an important strength surrogate) compared with the sham. Trabecular deterioration at L5 in OVx control was more severe than the appendicular sites as each of the connectivity and geometric measures was negatively affected. The endpoint micro-CT measurement in ASFW group in all cancellous bones was, by and large, comparable to E2 group, suggesting that ASF pretreatment afforded trabecular preservation after OVx equivalent to that of E2 supplementation. Furthermore, better architectural indices in the trabecular rich vertebra in the ASFW group translated to greater resistance to compressive failure in the lumbar vertebra, thereby implying that ASF supplementation during the period of peak bone gain could effectively reduce the risk of osteoporotic compressive fracture in postmenopausal women.

Better cortical parameters at baseline appear to have contributed in better cortical thickness and cortical bone area in the femur of ASFW group compared to OVx + veh. In fact, the cortical parameters in ASFW were comparable to the sham group. Interestingly, MMI in the diaphysis was increased after OVx, which was similar to a previous report [40] but contrary to significant decreases [41], and no changes were reported by others [42]. Dynamic histology evaluating MAR and BFR/BS showed uncoupled remodeling in the OVx group evident from reduced values compared to sham, which could thus favor net bone loss due to enhanced resorption upon E2 deficiency. By contrast, ASFW group had increased MAR and BFR/BS over OVx control and OVx + E2 groups, suggesting preservation of coupled remodeling by augmenting osteoblast function despite increased osteoclast activity due to OVx. Superior cortical deposition appeared to have conferred more strength in bending, as ASFW group exhibited greater elastic and strength properties over the OVx control, and the values were comparable to sham.

E2 deficiency is characterized by high turnover bone loss and OCN and CTx serve as surrogates for monitoring the efficacy of treatment of osteoporosis in both clinical and preclinical studies [43]. E2 supplementation to OVx rats strongly suppressed both markers and kept close to the sham levels. In the ASFW group, CTx was better suppressed than OCN. Because there is a good correlation between serum OCN and histomorphometric indices of bone formation [44], high serum OCN level in the ASFW group reflects the higher activity of the osteoblasts over the OVx + E2 group. Thus, ASFW group appears to simultaneously manifest an anti-catabolic and an osteogenic state, together contributing to its bone conserving effect after OVx.

## 5. Conclusion

In conclusion, our studies in the preclinical setting in female rats provide evidence that ASF supplementation during the skeletal growth and maturity could enhance peak bone mass and bestow greater bone conserving efficacy after OVx despite treatment withdrawal. ASF supplementation to young girls for an extended period may provide an effective preventive strategy for decreasing the risk of developing osteoporosis and fragility fracture after menopause.

## Figures and Tables

**Table 1:** Effect on body weight, antioxidant status, bone mass, and microarchitecture in growing female rats after 12 weeks of treatment (baseline).

Parameters	Vehicle (1% gum-acacia)	ASF (100 mg/kg/d)
Body weight (g)	201 ± 4.58	216.1 ± 9.68
Total antioxidant status (mmol/L)	1.44 ± 0.05	2.27 ± 0.07^b^
Bone length		
Femur (cm)	2.43 ± 0.02	2.57 ± 0.02^b^
Tibia (cm)	2.69 ± 0.03	2.91 ± 0.06^c^
Growth plate height		
Femur (*μ*m)	241.77 ± 2.23	264.9 ± 3.81^a^
Tibia (*μ*m)	148.07 ± 2.73	161.97 ± 3.94^c^
BMD (g HA/cm^3^)		
Femur diaphysis	1.02 ± 0.007	1.071 ± 0.004^a^
Femur metaphysis	0.23 ± 0.007	0.298 ± 0.01^b^
Tibia diaphysis	1.01 ± 0.01	1.067 ± 0.01^c^
Proximal tibia metaphysis	0.19 ± 0.01	0.257 ± 0.01^b^
Micro-CT measurements at femur cortical bone		
B.Ar (mm^2^)	4.28 ± 0.09	4.68 ± 0.11^c^
Ct.Th (mm)	0.21 ± 0.01	0.26 ± 0.05^c^
T.Ar (mm^2^)	9.88 ± 0.35	10.22 ± 0.55
Ma.Ar (mm^2^)	6.02 ± 0.39	5.53 ± 0.45
MMI (mm^4^)	10.21 ± 0.5	11.76 ± 0.58^c^
Micro-CT measurements at femur cancellous bone		
BV/TV (%)	15.8 ± 0.49	20.8 ± 0.61^a^
Tb.N (1/mm)	1.74 ± 0.05	2.252 ± 0.08^a^
Tb.Th (mm)	0.08 ± 0.002	0.089 ± 0.002
Conn.D (1/mm^3^)	157.37 ± 11.83	229.8 ± 14.81^a^
Tb.sp (mm)	0.48 ± 0.01	0.33 ± 0.03^b^
Tb.pf (1/mm)	3.45 ± 0.13	1.415 ± 0.11^a^
SMI	1.96 ± 0.05	1.68 ± 0.02^b^
DA	1.91 ± 0.01	1.892 ± 0.02
Micro-CT measurements at tibial cancellous bone		
BV/TV (%)	9.6 ± 0.81	15.3 ± 1.33^b^
Tb.N (1/mm)	1.28 ± 0.04	1.87 ± 0.15^b^
Tb.Th (mm)	0.08 ± 0.001	0.08 ± 0.001
Conn.D (1/mm^3^)	103.66 ± 12.72	166.36 ± 13.6^b^
Tb.sp (mm)	0.51 ± 0.03	0.32 ± 0.02^b^
Tb.pf (1/mm)	17.75 ± 1.78	12.56 ± 1.09^c^
SMI	2.26 ± 0.05	1.89 ± 0.02^a^
DA	2.11 ± 0.02	2.16 ± 0.08

Values represent mean ± SEM; *n* = 45 rats in vehicle group and *n* = 15 rats in ASF group. ^a^ *P* < 0.001, ^b^ *P* < 0.01, and ^c^ *P* < 0.05 compared to vehicle.

BMD: bone mineral density, B.Ar: bone area, Ct.Th.: cortical thickness, T.Ar: periosteal area, MMI: mean polar moment of inertia, Ma.Ar: marrow area, BV/TV: percent bone volume, Tb.N: trabecular number, Tb.Th: trabecular thickness, Conn.D: connection density, Tb.sp: trabecular separation, Tb.pf: trabecular pattern factor, SMI: structure model index, DA: degree of anisotropy.

**Table 2:** Effect of 8-week treatment withdrawal and ovariectomy on trabecular bone microarchitecture among various groups.

Parameters	OVx + vehicle (1% gum acacia)	OVx + vehicle (1% gum cacia) (ASFW)	OVx + E2 (10 *μ*g/kg/day)
Femoral cancellous bone			
BV/TV	−48.7 ± 0.6***	−17.6 ± 0.33^∗,p^	−20.2 ± 0.65^∗,p,f^
Tb.N	−50.9 ± 5.28***	−20.8 ± 2.14^∗,p^	−34.08 ± 2.72^∗∗,q,g^
Tb.Th	−5.6 ± 1.05	−2.5 ± 1.99	−1.8 ± 0.85
Conn.D	−60.8 ± 7.35***	−40.3 ± 5.53**	−50.08 ± 3.03**
Tb.sp	135.4 ± 10.57***	81.8 ± 8.84^∗∗,q^	118.3 ± 10.83^∗∗∗,g^
Tb.pf	338.8 ± 17.8***	49.4 ± 4.41^∗∗,p^	120.3 ± 3.85^∗∗∗,p,e^
SMI	11.5 ± 1.42*	2.32 ± 0.22^p^	1.9 ± 0.2^p^
DA	1.5 ± 0.05	2.7 ± 0.17^r^	1.9 ± 0.43
Tibial cancellous bone			
BV/TV	−51.7 ± 5.37***	−21.5 ± 2.08^∗,p^	−25.8 ± 3.92^∗,p^
Tb.N	−65.5 ± 2.45***	−20.8 ± 3.67^∗,p^	−17.4 ± 1.03^∗,p^
Tb.Th	−2.4 ± 0.01	−2.2 ± 0.013^p^	−5.1 ± 0.01^p,e^
Conn.D	−71.1 ± 2.57***	−50.3 ± 2.94^∗∗,p^	−42.3 ± 4.01^∗∗,p^
Tb.sp	192.5 ± 10.61***	48.5 ± 4.1^∗∗,p^	92.4 ± 7.14^∗∗∗,p,f^
Tb.pf	73.7 ± 4.83***	10.2 ± 3.29^p^	28.9 ± 5.28^∗,p,g^
SMI	21.6 ± 2.57*	1.7 ± 0.73^p^	2.4 ± 0.9^p^
DA	4.04 ± 1.01	3.2 ± 0.86	5.4 ± 1.42

All parameters expressed in terms of mean percentage change from treatment termination (baseline). Values are mean ± SEM from 15 rats/group. ***P < 0.00, **P < 0.01, and *P < 0.05 compared to baseline. Student's *t*-test (unpaired) was used for intragroup statistics.

^p^ *P* < 0.001, ^q^ *P* < 0.01, and ^r^ *P* < 0.05 compared to OVx + vehicle; ^e^ *P* < 0.001, ^f^ *P* < 0.01, and ^g^ *P* < 0.05 compared to ASFW.

One-way ANOVA was followed for intergroup statistics.

BV/TV: percent bone volume, Tb.N: trabecular number, Tb.Th: trabecular thickness, Conn.D: connection density, Tb.sp: trabecular separation, Tb.pf: trabecular pattern factor, SMI: structure model index, DA: degree of anisotropy.

**Table 3:** Effect on body weight, bone biochemical markers, trabecular bone mass, microarchitecture, and strength following ovariectomy and treatment withdrawal at the endpoint.

Parameters	Sham + vehicle (1% gum acacia)	OVx + vehicle (1% gum acacia)	OVx + vehicle (1% gum acacia) (ASFW)	OVx + E2 (10 *μ*g/kg/day)
Body weight (gm)	232.3 ± 8.89	303.1 ± 8.27^p^	297.4 ± 7.93^p^	276.7 ± 13.05^r^
Bone biochemical markers				
Serum OCN (ng/mL)	105.49 ± 8.76^p^	244.96 ± 6.76	203.93 ± 9.84^q,a^	97.27 ± 9.99^p,e^
Urinary CTx (ng/mL)	46.75 ± 11.4^p^	113.69 ± 4.66	76.6 ± 3.23^p,b^	66.37 ± 3.24^p,c^
BMD (g HA/cm^3^)				
Femur metaphysis	0.234 ± 0.01^q^	0.166 ± 0.001	0.198 ± 0.007^r^	0.185 ± 0.01^r^
Proximal tibia metaphysis	0.248 ± 0.01^p^	0.118 ± 0.006	0.17 ± 0.006^q,a^	0.171 ± 0.01^q,a^
Vertebral cancellous bone	0.31 ± 0.014^p^	0.219 ± 0.006	0.264 ± 0.007^q,b^	0.265 ± 0.009^q,b^
Micro-CT measurements at femur cancellous bone				
BV/TV (%)	24.8 ± 1.87^p^	12.07 ± 0.12	17.12 ± 0.38^p,a^	14.12 ± 0.56^r,a,f^
Tb.N (1/mm)	2.67 ± 0.27^p^	1.27 ± 0.01	1.79 ± 0.06^r,a^	1.71 ± 0.05^r,a^
Tb.Th (mm)	0.09 ± 0.0004	0.09 ± 0.001	0.09 ± 0.001	0.09 ± 0.002
Conn.D (1/mm^3^)	158.66 ± 11.79^p^	58.48 ± 3.6	82.37 ± 3.76^r,a^	72.15 ± 4.78^a^
Tb.sp (mm)	0.3 ± 0.03^p^	0.73 ± 0.04	0.65 ± 0.01^a^	0.59 ± 0.02^r,a^
Tb.pf (1/mm)	1.42 ± 0.51^p^	6.33 ± 0.3	2.44 ± 0.27^p^	3.39 ± 0.4^p,c^
SMI	1.48 ± 0.07^q^	1.7 ± 0.03	1.43 ± 0.02^q^	1.5 ± 0.04^q^
DA	1.8 ± 0.03	1.82 ± 0.06	1.8 ± 0.06	1.69 ± 0.02
Micro-CT measurements at tibial cancellous bone				
BV/TV (%)	20.35 ± 1.64^p^	8.8 ± 0.65	13.38 ± 0.64^r,a^	13.67 ± 1.06^r,a^
Tb.N (1/mm)	2.32 ± 0.17^p^	0.98 ± 0.07	1.36 ± 0.06^r,a^	1.45 ± 0.09^r,a^
Tb.Th (mm)	0.08 ± 0.001	0.08 ± 0.001	0.09 ± 0.001	0.09 ± 0.001
Conn.D (1/mm^3^)	137 ± 10.12^p^	33.9 ± 2.12	55.52 ± 6.32^r,a^	59.42 ± 4.15^r,a^
Tb.sp (mm)	0.23 ± 0.01^p^	0.69 ± 0.04	0.5 ± 0.02^p,a^	0.46 ± 0.01^p,a^
Tb.pf (1/mm)	6.41 ± 0.35^q^	12.23 ± 0.78	8.19 ± 0.48^q^	8.29 ± 0.48^q^
SMI	1.74 ± 0.03^p^	2.05 ± 0.05	1.84 ± 0.02^q^	1.83 ± 0.05^q^
DA	2.16 ± 0.04	2.18 ± 0.05	2.006 ± 0.03	2.01 ± 0.03
Micro-CT measurements at vertebral cancellous bone				
BV/TV (%)	29.96 ± 1.85^p^	15.06 ± 1.36	21.06 ± 0.63^r,b^	22.24 ± 3.25^r,c^
Tb.N (1/mm)	2.003 ± 0.1^q^	1.28 ± 0.14	1.61 ± 0.07	1.62 ± 0.19
Tb.Th (mm)	0.13 ± 0.005^p^	0.102 ± 0.001	0.12 ± 0.001^p,c^	0.11 ± 0.002^p,b^
Conn.D (1/mm^3^)	64.99 ± 4.23^p^	17.77 ± 1.56	26.35 ± 2.16^r,a^	28.83 ± 1.12^r,a^
Tb.sp (mm)	0.38 ± 0.009^q^	0.49 ± 0.02	0.41 ± 0.01^r^	0.41 ± 0.02^r^
Tb.pf (1/mm)	0.86 ± 0.76^r^	6.37 ± 0.82	4.16 ± 0.48	4.49 ± 1.22
SMI	0.86 ± 0.11^p^	1.44 ± 0.04	0.96 ± 0.09^p^	1.19 ± 0.01^r,b,g^
DA	2.09 ± 0.07^p^	3.57 ± 0.09	2.62 ± 0.11^p,b^	2.7 ± 0.15^p,b^
Vertebral bone strength				
Ultimate load (N)	203.33 ± 16.99^p^	92 ± 4.32	172 ± 17.65^p^	143.33 ± 4.71^q,b^
Energy (mJ)	1009.05 ± 104.5^q^	544.27 ± 83.01	936.88 ± 43.47^q^	1031.44 ± 112.01^q^
Stiffness (N/mm)	268.03 ± 41.77^p^	66.57 ± 13.67	206.86 ± 18.93^q^	252.86 ± 40.6^q^

Values represent mean ± SEM; *n* = 15 rats/group. ^p^ *P* < 0.001, ^q^ *P* < 0.01, and ^r^ *P* < 0.05 compared to OVx + vehicle; ^a^ *P* < 0.001, ^b^ *P* < 0.01, and ^c^ *P* < 0.05 compared to sham; ^f^ *P* < 0.01 and ^g^ *P* < 0.05 compared to ASFW.

BMD: bone mineral density, BV/TV: percent bone volume, Tb.N: trabecular number, Tb.Th: trabecular thickness, Conn.D: connection density, Tb.sp: trabecular separation, Tb.pf: trabecular pattern factor, SMI: structure model index, DA: degree of anisotropy.

**Table 4:** Effect on bone formation indices, microarchitecture, and strength at femur diaphysis following ovariectomy and treatment withdrawal at the endpoint.

Parameters	Sham + vehicle (1% gum acacia)	OVx + vehicle (1% gum acacia)	OVx + vehicle (1% gum acacia) (ASFW)	OVx + E2 (10 *μ*g/kg/day)
Bone formation indices at femur mid-diaphysis				
pMS/BS (%)	96.96 ± 0.5^p^	85.92 ± 2.72	95.43 ± 0.46^q^	89.87 ± 1.86^c,g^
pMAR (*μ*m/day)	0.43 ± 0.01^p^	0.2 ± 0.01	0.34 ± 0.01^p,a^	0.23 ± 0.001^a,e^
pBFR /BS (*μ*m^3^/*μ*m^2^/yr)	3.79 ± 0.05^p^	1.87 ± 0.08	3.13 ± 0.13^p,b^	2.05 ± 0.16^a,e^
Micro-CT measurements at femur mid-diaphysis				
B.Ar (mm^2^)	4.7 ± 0.14^q^	3.79 ± 0.06	4.53 ± 0.06^q^	4.62 ± 0.21^q^
Ct.Th (mm)	0.49 ± 0.01^r^	0.35 ± 0.02	0.47 ± 0.02^r^	0.4 ± 0.03
T.Ar (mm^2^)	8.16 ± 0.05	8.19 ± 0.21	8.13 ± 0.1	8.25 ± 0.6
Ma.Ar (mm^2^)	3.37 ± 0.06^r^	4.66 ± 0.26	3.45 ± 0.13^q^	3.77 ± 0.32^r^
MMI (mm^4^)	7.33 ± 0.61^r^	10.2 ± 0.69	7.63 ± 0.37^r^	8.41 ± 0.65
Bone strength at femur mid-diaphysis				
Ultimate load (N)	142.66 ± 10.5^p^	101.33 ± 4.27	161.66 ± 5.49^p^	149.75 ± 1.88^q^
Energy (mJ)	70.66 ± 2.45^q^	42.65 ± 3.85	82.91 ± 6.52^q^	65.241 ± 1.16^r^
Stiffness (N/mm)	247.62 ± 13.39^p^	133.58 ± 20.91	252.475 ± 13.12^p^	248.161 ± 22.41^q^

Values represent mean ± SEM; *n* = 15 rats/group. ^p^ *P* < 0.001, ^q^ *P* < 0.01, and ^r^ *P* < 0.05 compared to OVx + vehicle; ^a^ *P* < 0.001, ^b^ *P* < 0.01, and ^c^ *P* < 0.05 compared to sham; ^e^ *P* < 0.001 and ^g^ *P* < 0.05 compared to ASFW.

pMS/BS: periosteal mineralizing surface per bone surface, pMAR: periosteal mineral apposition rate, pBFR/BS: periosteal bone formation rate/bone surface. B.Ar: bone area, Ct.Th.: cortical thickness, T.Ar: periosteal area, MMI: mean polar moment of inertia, Ma.Ar: medullary area.
